# Particulate Matter and Subclinical Atherosclerosis: Associations between Different Particle Sizes and Sources with Carotid Intima-Media Thickness in the SAPALDIA Study

**DOI:** 10.1289/EHP161

**Published:** 2016-06-03

**Authors:** Inmaculada Aguilera, Julia Dratva, Seraina Caviezel, Luc Burdet, Eric de Groot, Regina E. Ducret-Stich, Marloes Eeftens, Dirk Keidel, Reto Meier, Laura Perez, Thomas Rothe, Emmanuel Schaffner, Arno Schmit-Trucksäss, Ming-Yi Tsai, Christian Schindler, Nino Künzli, Nicole Probst-Hensch

**Affiliations:** 1 Swiss Tropical and Public Health Institute, Basel, Switzerland; 2 University of Basel, Basel, Switzerland; 3 Hôpital Intercantonal de la Broye, Payerne, Switzerland; 4 Imagelabonline and Cardiovascular, Eindhoven and Lunteren, the Netherlands; 5 Zürcher Höhenklinik Davos, Davos Clavadel, Switzerland; 6 Division Sports and Exercise Medicine, Department of Sport, Exercise and Health, University of Basel, Basel, Switzerland; 7 Department of Environmental & Occupational Health Sciences, University of Washington, Seattle, Washington, USA

## Abstract

**Background:**

Subclinical atherosclerosis has been associated with long-term exposure to particulate matter (PM), but the relevance of particle size and sources of exposure remains unclear.

**Objectives:**

We investigated the association of long-term exposure to PM10 (≤ 10 μm), PM2.5 (≤ 2.5 μm: total mass, vehicular, and crustal sources), and ultrafine particles [UFP < 0.1 μm: particle number concentration (PNC) and lung-deposited surface area (LDSA)] with carotid intima-media thickness (CIMT).

**Methods:**

We used data from 1,503 participants ≥ 50 years old who participated in the third examination of the Swiss SAPALDIA cohort. Exposures were obtained from dispersion models and land-use regression models. Covariate information, including previous cardiovascular risk factors, was obtained from the second and third SAPALDIA examinations.

**Results:**

The adjusted percent difference in CIMT associated with an exposure contrast between the 10th and 90th percentile was 1.58% (95% CI: –0.30, 3.47%) for PM10, 2.10% (95% CI: 0.04, 4.16%) for PM2.5, 1.67% (95% CI: –0.13, 3.48%) for the vehicular source of PM2.5, –0.58% (95% CI: –3.95, 2.79%) for the crustal source of PM2.5, 2.06% (95% CI: 0.03, 4.10%) for PNC, and 2.32% (95% CI: 0.23, 4.40%) for LDSA. Stronger associations were observed among diabetics, subjects with low-educational level, and those at higher cardiovascular risk.

**Conclusions:**

CIMT was associated with exposure to PM10, PM2.5, and UFP. The PM2.5 source-specific analysis showed a positive association for the vehicular source but not for the crustal source. Although the effects of PNC and LDSA were similar in magnitude, two-pollutant and residual-based models suggested that LDSA may be a better marker for the health relevance of UFP.

**Citation:**

Aguilera I, Dratva J, Caviezel S, Burdet L, de Groot E, Ducret-Stich RE, Eeftens M, Keidel D, Meier R, Perez L, Rothe T, Schaffner E, Schmit-Trucksäss A, Tsai MY, Schindler C, Künzli N, Probst-Hensch N. 2016. Particulate matter and subclinical atherosclerosis: associations between different particle sizes and sources with carotid intima-media thickness in the SAPALDIA study. Environ Health Perspect 124:1700–1706; http://dx.doi.org/10.1289/EHP161

## Introduction

Epidemiological and experimental research has provided sufficient evidence for a causal effect of ambient air pollution [mainly particulate matter (PM)] on cardiovascular mortality and morbidity (Brook et al. 2010). These studies suggest that air pollution not only triggers acute cardiovascular events in the short term, but also influences the development of underlying chronic cardiovascular pathologies, atherosclerosis being the major one (Künzli et al. 2011). Experimental animal studies provide strong evidence for a causal atherogenic role of air pollution through oxidative stress and systemic inflammation (Araujo et al. 2008; Soares et al. 2009; Sun et al. 2005).

In the last decade, a growing number of cross-sectional studies have assessed the relationship between air pollution and subclinical atherosclerosis in adult populations, with carotid intima-media thickness (CIMT) being the most frequently used indicator. However, findings were not consistent (Bauer et al. 2010; Diez Roux et al. 2008; Kim et al. 2014; Künzli et al. 2005; Perez et al. 2015; Rivera et al. 2013; Su et al. 2015; Wilker et al. 2013). Four longitudinal studies published to date, all of them conducted in North America, also yielded inconsistent results. For example, two of them reported an association of PM_2.5_ and proximity to highways with CIMT progression (Adar et al. 2013; Künzli et al. 2010), the other two did not find an effect of any pollutant (Wilker et al. 2013; Gan et al. 2014). A recent meta-analysis of those studies that assessed PM_2.5_ exposure showed a positive association between CIMT and PM_2.5_, both cross sectionally and longitudinally (Provost et al. 2015).

There is a need to assess the extent to which the cardiovascular effects of ambient PM mass vary by particle type and composition (Araujo et al. 2008; Brook et al. 2010). The association between specific PM_2.5_ components and subclinical atherosclerosis has only been investigated in the Multi-Ethnic Study of Atherosclerosis (MESA) study (Kim et al. 2014). To our knowledge, no epidemiological study has assessed the association of subclinical atherosclerosis with ultrafine particles (UFP), despite their stronger atherogenic effect in animal studies when compared to larger size fractions (Araujo et al. 2008). In addition, toxicological studies suggest that the particle surface area is superior to particle mass for evaluating the inflammatory potential of UFP. This makes lung-deposited surface area (LDSA) a promising metric for investigating health effects of UFP (Wittmaack 2007). However, the added value of this metric has not yet been assessed epidemiologically.

The aim of the present study was to examine the cross-sectional association of long-term home outdoor exposures to different sizes of PM: PM_10_ (≤ 10 μm in aerodynamic diameter), PM_2.5_ (≤ 2.5 μm in aerodynamic diameter), and UFP (< 0.1 μm in aerodynamic diameter) and different sources of PM_2.5_ (vehicular and crustal) with CIMT measured in the third examination of the Swiss Cohort Study on Air Pollution and Lung and Heart Diseases in Adults (SAPALDIA). In addition, we investigated the potential effect modification by cardiovascular risk factors assessed in the previous SAPALDIA health examination. Finally, we also compared the results obtained with two advanced CIMT reading protocols (static and dynamic).

## Methods

### Study Population

SAPALDIA is a population-based cohort study conducted in eight geographic areas in Switzerland. The present analysis focuses on the four study areas where PM and UFP measurements were performed: Basel, Geneva, Lugano, and Wald. In these four areas, the study started in 1991 with a random sample of 5,318 participants who underwent a detailed health examination (Martin et al. 1997). Two follow-up assessments were conducted in 2001–2002 (SAPALDIA2) and in 2010–2011 (SAPALDIA3). In SAPALDIA2, health assessments were repeated in 4,489 participants and included blood testing for cardiometabolic biomarkers (Ackermann-Liebrich 2005). In SAPALDIA3, CIMT was measured in 1,785 out of the 2,502 participants **≥** 50 years old at the time of examination. SAPALDIA complies with the Declaration of Helsinki and ethical approval was granted by the respective Swiss cantonal ethical committees. Study participants gave written informed consent.

For the present analysis, we restricted the sample to participants of both SAPALDIA2 and SAPALDIA3 with valid CIMT and air pollution measurements as well as complete information in relevant covariates (*n* = 1,503).

### CIMT Measurements

Carotid ultrasound measurements were conducted under the supervision and technical assistance of Imagelabonline & Cardiovascular (Eindhoven and Lunteren, Netherlands), and the Department of Sports, Exercise and Health of the University of Basel (DSBG). Measurements by trained and certified field workers followed a standardized imaging protocol using standardized ultrasound instruments (UF-870 machine LA38 linear array transducer, Fukuda Denshi, Japan). Both left and right common carotid arterial (CCA) far wall were visualized in two predefined angles (longitudinal ear-to-ear view and horizontal view). These methods have been described elsewhere (Caviezel et al. 2013; Teynor et al. 2012).

Field workers saved end-diastolic DICOM B-mode still images (static reading method) and sequential images (NATIVE clips, dynamic reading method). For both reading methods, CIMT was calculated as the average of the four mean CIMT measures obtained for each individual. We focused the analysis on the mean CIMT measurements obtained using the NATIVE clips from DSBG. More details about CIMT measurements are given in “Additional methodological details, CIMT measurements” in the Supplemental Material.

### Air Pollution Exposure Assessment

PM_10_, PM_2.5_, particle number concentration (PNC), and LDSA were measured between 2011 and 2012 in four SAPALDIA areas: Basel, Geneva, Lugano, and Wald (Eeftens et al. 2015; Meier et al. 2015). Measurements of PNC and LDSA were conducted with miniature diffusion size classifiers (miniDiSC) (Fierz et al. 2011), measuring particles between 10 and 300 nm, with a flow rate of 1.0 L/min. LDSA is defined as the particle surface area concentration per unit volume of air, weighted by the deposition probability in the lung. The deposition probability is customarily calculated according to the ICRP report 66 (ICRP 1994). Light absorbance (a marker of combustion-derived PM) and trace element concentrations were measured on PM_2.5_ filters, and a principal-component factor analysis was performed to identify three main sources of PM_2.5_ constituents: vehicular, crustal, and long-range transport (Aguilera et al. 2015). Then, land-use regression (LUR) models were developed to assess the spatial distribution of PNC, LDSA, and the factor scores of both the vehicular and crustal PM_2.5_ sources across the four study areas. The spatial variation explained by the LUR models, evaluated by the adjusted coefficient of determination (*R* ^2^), was high for PNC (*R* ^2^ = 0.85), LDSA (*R* ^2^ = 0.89) and the vehicular source of PM_2.5_ (*R* ^2^ = 0.76), and moderate for the crustal source of PM_2.5_ (*R* ^2^ = 0.46) (Aguilera et al. 2015). Models were then applied to assign bi-annual exposures (corresponding to the 2011–2012 period) to the participants, using the residential addresses reported at the SAPALDIA3 examination.

Exposure estimates of PM_10_ and PM_2.5_ were obtained from the PolluMap Gaussian dispersion model (Swiss Agency for the Environment, Forests and Landscape 2003; Swiss Federal Office for the Environment 2013), used in previous SAPALDIA health analyses (Downs et al. 2007; Eze et al. 2014) and available on a grid with a spatial resolution of 200 × 200 m. This option was preferred over models built from SAPALDIA measurements, since the availability of dispersion models for the years 2000, 2005, and 2010, and the interpolation models for the remaining years, allowed us to estimate a time-weighted average residential PM_10_ exposure between SAPALDIA2 and SAPALDIA3 examinations (mean ± SD = 8.4 ± 0.4 years). For each study participant, the average exposure was calculated using the concentrations at the residential address history between SAPALDIA2 and SAPALDIA3, weighted by the percentage of the time period spent at each address. Equivalent time-weighted average exposures were also obtained for PM_2.5._ They were also based on PM_2.5_ emission data for the years 2000, 2005, and 2010. Due to the lack of official interpolation models of PM_2.5_ for the intermediate years, we estimated PM_2.5_ levels for these years assuming relative annual fluctuations around linear trends to be identical for PM_10_ and PM_2.5_, given the high spatial correlation between PM_2.5_ and PM_10_ observed in the air quality monitoring stations across the country for the period 2000–2012 (*R* ^2^ = 0.93).

In addition to the time-weighted average exposure between SAPALDIA2 and SAPALDIA3, we also estimated the average PM_10_ and PM_2.5_ exposures for a more recent exposure period (i.e., the 365 days before the CIMT assessment date). This additional metric was mainly chosen to allow for a direct comparison with previous studies, as well as for comparison with the longer-term exposure between SAPALDIA2 and SAPALDIA3 that relied on a larger proportion of interpolated data. Furthermore, investigating associations with more recent exposure is of interest in the absence of a clear understanding of the latency of air pollution effects on CIMT.

### Statistical Analysis

Mixed linear models with a random intercept for the different study areas were fitted to estimate the percent change in CIMT associated with an interdecile range (10th to 90th percentile) increase in air pollution exposures. For PM_10_ and PM_2.5_, associations are also reported for a 10 μg/m^3^ increase (see Table S5 and Figure S3) to allow for comparison of associations with exposures between SAPALDIA2 and SAPALDIA3 and exposures during the last year before the CIMT assessment. A set of six models were fitted in a staged manner to investigate the co-varying effects of several covariates, including potential confounders and potential intermediates or clinical correlates of CIMT. After a crude model (model 1), a minimally adjusted model included sex, age, and sex–age interaction (model 2). The main model added educational level, smoking status, pack-years of cigarettes smoked between SAPALDIA2 and SAPALDIA3, and body mass index (BMI) in both SAPALDIA2 and SAPALDIA3 (model 3). Further adjustments added lifestyle variables (exposure to environmental tobacco smoke, alcohol intake, and physical activity in SAPALDIA3) in model 4, and biomarkers and medical variables [diabetes, high-density lipoprotein (HDL) cholesterol, and creatinine in SAPALDIA2; and systolic blood pressure, intake of antihypertensive and lipid-lowering medication in SAPALDIA3] in model 5. We also considered traffic noise as a potential confounder and fitted a sixth model adjusted for the annual average of night-time traffic noise, obtained from the Swiss SonBase model and only available for the residential addresses at SAPALDIA2.

A set of predetermined variables was tested for effect modification, namely sex, age, BMI, educational level, smoking status, moderate physical activity, cardiovascular disease (CVD), diabetes, chronic obstructive pulmonary disease (COPD), medication intake (antihypertensive and lipid lowering), and CVD risk at SAPALDIA2 [estimated using the European Society of Cardiology heart risk score (SCORE) algorithm for a 10-year risk of a fatal event] (Conroy et al. 2003). Given the stronger atherogenic effects of UFP in animal studies, we also fitted a two-pollutant model adjusted for PNC.

Additional analyses were performed to *a*) estimate the odds ratio (OR) of having CIMT above the 75th percentile of the cohort-specific predicted value, since the upper tail of the CIMT distribution has particular relevance in clinical settings; *b*) investigate the potential exposure misclassification related to residential mobility between SAPALDIA2 and SAPALDIA3; and *c*) compare CIMT measurements from dynamic and static reading methods.

All statistical analyses were performed using STATA (version 12.1; Stata Corp., College Station, TX, USA). *p*-Values below 0.05 defined statistical significance. More details on covariate information and statistical analyses are given in “Additional methodological details, Statistical analysis” in the Supplemental Material.

## Results

Subjects included in the analysis were slightly younger, had higher educational status, lower BMI and lower prevalence of CVD and diabetes than those excluded from the analysis according to the previously defined criteria (see Table S1). The mean (± SD) follow-up time between SAPALDIA2 and SAPALDIA3 was 8.4 ± 0.4 years for the participants included in the analysis (Table 1). Participants were between 50 and 81 years old, with a mean of 63.9 ± 8.2 years. There were some statistically significant differences between movers and nonmovers, mostly driven by the younger average age of movers (Table 1).

**Table 1:** Characteristics of the study population in SAPALDIA2 (S2) and SAPALDIA3 (S3).

Characteristics	Total (*n *= 1,503)	Movers (*n *= 401)	Nonmovers (*n *= 1,102)	*p*-Value^*a*^
Study area				0.06
Basel	22.0	21.9	22.1
Geneva	14.3	11.0	15.5
Lugano	25.7	29.4	24.3
Wald	38.0	37.7	38.1
Follow-up time between S2 and S3 (years)	8.4 ± 0.4	8.4 ± 0.5	8.4 ± 0.4	0.27
Mean CIMT in S3 (mm)	0.74 ± 0.13	0.73 ± 0.13	0.75 ± 0.13	0.01
Average PM_10_ between S2 and S3 (μg/m^3^)	23.2 ± 3.8	23.1 ± 3.9	23.2 ± 3.7	0.32
Average PM_10_ of the last year^*b*^ (μg/m^3^)	20.2 ± 2.3	19.9 ± 2.5	20.2 ± 2.2	0.02
Average PM_2.5_ between S2 and S3 (μg/m^3^)	17.0 ± 2.0	16.9 ± 2.1	17.0 ± 2.0	0.23
Average PM_2.5_ of the last year^*b*^ (μg/m^3^)	15.2 ± 1.6	15.1 ± 1.7	15.2 ± 1.5	0.05
Vehicular source of PM_2.5_, biennial average^*c*^ (score)	–0.6 ± 0.9	–0.7 ± 0.8	–0.5 ± 0.9	< 0.001
Crustal source of PM_2.5_, biennial average^*c*^ (score)	–0.3 ± 0.6	–0.4 ± 0.6	–0.3 ± 0.6	< 0.001
PNC, biennial average^*c*^ (particles/cm^3^)	11,184 ± 4,862	10,596 ± 4,557	11,385 ± 4,948	0.007
LDSA, biennial average^*c*^ (μm^2^/cm^3^)	30.8 ± 11.5	30.3 ± 11.5	31.0 ± 11.6	0.29
Night-time traffic noise in S2, dB(A)	38.8 ± 7.7	38.4 ± 7.4	38.9 ± 7.9	0.15
Age (years)	63.9 ± 8.2	61.7 ± 7.5	64.8 ± 8.3	< 0.001
Women	53.0	52.9	53.1	0.94
Educational status				0.36
Low (primary education)	5.5	4.2	5.9
Middle (secondary or vocational education)	64.2	66.3	63.4
High (technical college or university)	30.3	29.4	30.7
Smoking status in S2				0.14
Never smoker	43.6	43.4	43.7
Former smoker	34.1	31.2	35.2
Current smoker	22.2	25.4	21.1
Smoking pack-years from S2 to S3	1.12 ± 2.95	1.43 ± 3.37	1.01 ± 2.78	0.01
Exposed to ETS in the last year^*b*^	12.8	16.5	11.5	0.01
Alcohol intake in S3, several times per week	45.9	43.9	45.0	0.71
Moderate physical activity category change between S2 and S3				0.53
Inactive mantainer	26.5	27.6	26.2
Relapser	17.1	18.6	16.6
Adopter	21.7	19.4	22.6
Active mantainer	34.6	34.5	34.7
Moderate physical activity in S3, sufficiently active	56.0	52.7	57.2	0.12
BMI in S2 (kg/m^2^)	25.6 ± 4.0	25.6 ± 4.1	25.6 ± 4.0	0.44
BMI in S3 (kg/m^2^)	26.3 ± 4.4	26.2 ± 4.4	26.3 ± 4.3	0.74
Systolic blood pressure in S2 (mmHg)	127.8 ± 18.8	126.1 ± 18.4	128.4 ± 19.0	0.02
Systolic blood pressure in S3 (mmHg)	134.9 ± 18.9	133.2 ± 17.8	135.6 ± 19.2	0.04
Total cholesterol in S2 (mg/dL)	236.5 ± 41.4	235.3 ± 41.0	237.0 ± 41.6	0.31
HDL in S2 (mg/dL)	59.1 ± 17.4	59.9 ± 17.6	58.8 ± 17.3	0.29
Triglycerides in S2 (mg/dL)	155.1 ± 97.6	144.9 ± 84.9	158.9 ± 101.7	0.01
Creatinine in S2 (mg/dL)	0.99 ± 0.14	0.98 ± 0.14	0.99 ± 0.14	0.14
CVD risk SCORE^*d*^ in S2, 10-year risk ≥ 5%	5.7	4.5	6.2	0.23
Doctor-diagnosed CVD in S2	24.6	18.2	27.0	< 0.001
Diabetes in S2	3.9	3.7	3.9	0.89
COPD in S2	22.1	19.7	23.0	0.19
Antihypertensive medication in S3	33.1	29.3	34.5	0.06
Lipid modifier medication in S3	20.1	17.0	21.2	0.08
Note: The study characteristics are presented as percent or mean ± standard deviation. ^***a***^*p*-Value of the difference between movers and nonmovers using chi-square test or Mann–Withney test. ^***b***^Average of the 365 days before the CIMT examination date. ^***c***^Exposure estimated for the 2011–2012 period. Sources of PM_2.5_ are expressed as a score derived from principal-component factor analysis (Aguilera et al. 2015). ^***d***^Score for a 10-year risk of a fatal event (Conroy et al. 2003).				

Scatter plots of CIMT versus air pollution (see Figure S1) show little overlap between the area with the highest pollutant concentrations (Lugano) and the one with the lowest (Wald). The correlation among air pollution exposure estimates was high (see Table S2). Night-time traffic noise showed low correlation with air pollution exposure estimates (*r* = 0.17–0.26).

The association between CIMT and air pollution was positive across all exposure estimates in all models (Table 2). All crude associations clearly decreased after adjusting for sex, age, and sex–age interaction (model 2), and slightly increased in the main model (model 3) for all pollutants except the crustal source of PM_2.5_. Our findings were robust to further adjustment for lifestyle variables and potential intermediates (models 4 and 5): This pattern was independent of the decrease in sample size (data not shown). Associations among nonmovers showed the same pattern as in the whole study population across the different adjustment steps, except for the crustal source of PM_2.5_.

**Table 2:** Estimated percent change in CIMT (95% CI) associated with an interdecile range (10th to 90th percentile) increase in air pollution exposures, for the entire sample and for nonmovers.

Exposure	All subjects		Nonmovers	
	*n*	% change (95% CI)	*n*	% change (95% CI)
Average PM_10_ between S2 and S3 (increase of 10 μg/m^3^)	
Model 1 (crude)	1,491	3.98 (1.69, 6.26)	1,101	4.35 (1.64, 7.06)
Model 2^*a*^	1,491	1.97 (–0.06, 4.00)	1,101	2.06 (–0.34, 4.46)
Model 3^*a*^ (main)	1,491	2.33 (0.28, 4.38)	1,101	2.22 (–0.21, 4.66)
Model 4^*a*^	1,443	2.30 (0.22, 4.38)	1,063	2.12 (–0.34, 4.59)
Model 5^*a*^	1,340	2.76 (0.61, 4.91)	983	2.43 (–1.21, 6.06)
Model 6^*a*^		NA	983	2.61 (–1.11, 6.33)
Average PM_10_ of the last year^*b*^ (increase of 5.5 μg/m^3^)
Model 1 (crude)	1,500	3.05 (0.94, 5.15)	1,102	4.00 (1.44, 6.55)
Model 2^*a*^	1,500	1.38 (–0.48, 3.25)	1,102	2.18 (–0.08, 4.44)
Model 3^*a*^ (main)	1,500	1.58 (–0.30, 3.47)	1,102	2.22 (–0.07, 4.51)
Model 4^*a*^	1,452	1.61 (–0.30, 3.52)	1,064	2.24 (–0.08, 4.57)
Model 5^*a*^	1,348	1.83 (–0.16, 3.82)	984	2.96 (0.55, 5.37)
Model 6^*a*^		NA	984	3.43 (0.87, 5.99)
Average PM_2.5_ between S2 and S3 (increase of 5.6 μg/m^3^)	
Model 1 (crude)	1,491	4.49 (2.12, 6.87)	1,101	4.76 (1.94, 7.58)
Model 2^*a*^	1,491	2.31 (0.20, 4.42)	1,101	2.40 (–0.09, 4.90)
Model 3^*a*^ (main)	1,491	2.63 (0.50, 4.77)	1,101	2.58 (0.05, 5.11)
Model 4^*a*^	1,443	2.61 (0.45, 4.78)	1,063	2.49 (–0.07, 5.06)
Model 5^*a*^	1,340	3.06 (0.83, 5.30)	983	3.37 (0.70, 6.03)
Model 6^*a*^		NA	983	3.66 (0.91, 6.42)
Average PM_2.5_ of the last year^*b*^ (increase of 4.2 μg/m^3^)
Model 1 (crude)	1,500	3.99 (1.69, 6.29)	1,102	4.68 (1.91, 7.45)
Model 2^*a*^	1,500	1.92 (–0.12, 3.96)	1,102	2.52 (0.07, 4.98)
Model 3^*a*^ (main)	1,500	2.10 (0.04, 4.16)	1,102	2.57 (0.08, 5.06)
Model 4^*a*^	1,452	2.08 (0.00, 4.17)	1,064	2.50 (–0.02, 5.03)
Model 5^*a*^	1,348	2.43 (0.26, 4.60)	984	3.34 (0.72, 5.97)
Model 6^*a*^		NA	984	3.63 (0.92, 6.34)
Vehicular source of PM_2.5_, biennial average^*c*^
Model 1 (crude)	1,503	3.16 (1.13, 5.19)	1,102	3.47 (1.11, 5.84)
Model 2^*a*^	1,503	1.49 (–0.30, 3.28)	1,102	2.12 (0.04, 4.21)
Model 3^*a*^ (main)	1,503	1.67 (–0.13, 3.48)	1,102	2.26 (0.15, 4.36)
Model 4^*a*^	1,455	1.77 (–0.07, 3.60)	1,064	2.36 (0.22, 4.50)
Model 5^*a*^	1,351	2.11 (0.21, 4.00)	984	3.05 (0.84, 5.26)
Model 6^*a*^		NA	984	3.69 (1.28, 6.09)
Crustal source of PM_2.5_, biennial average^*c*^
Model 1 (crude)	1,503	1.41 (–1.87, 4.70)	1,102	3.32 (0.78, 5.86)
Model 2^*a*^	1,503	0.83 (–1.09, 2.75)	1,102	1.92 (–0.31, 4.16)
Model 3^*a*^ (main)	1,503	–0.58 (–3.95, 2.79)	1,102	1.91 (–0.34, 4.17)
Model 4^*a*^	1,455	–0.49 (–3.93, 2.96)	1,064	1.96 (–0.33, 4.24)
Model 5^*a*^	1,351	–1.81 (–5.30, 1.69)	984	2.40 (0.05, 4.76)
Model 6^*a*^		NA	984	2.93 (0.38, 5.47)
PNC, biennial average^*c*^ (increase of 12,639 particles/cm^3^)	
Model 1 (crude)	1,449	3.47 (1.20, 5.74)	1,080	3.44 (0.84, 6.05)
Model 2^*a*^	1,449	1.63 (–0.38, 3.64)	1,080	1.78 (–0.51, 4.08)
Model 3^*a*^ (main)	1,449	2.06 (0.03, 4.10)	1,080	1.98 (–0.35, 4.30)
Model 4^*a*^	1,402	2.13 (0.05, 4.20)	1,042	2.05 (–0.31, 4.42)
Model 5^*a*^	1,302	2.90 (0.75, 5.05)	964	3.04 (0.58, 5.51)
Model 6^*a*^		NA	964	3.58 (0.93, 6.23)
LDSA, biennial average^*c*^ (increase of 30.5 μm^2^/cm^3^)
Model 1 (crude)	1,449	3.67 (1.35, 5.98)	1,080	3.83 (1.14, 6.52)
Model 2^*a*^	1,449	1.86 (–0.19, 3.90)	1,080	2.02 (–0.35, 4.39)
Model 3^*a*^ (main)	1,449	2.32 (0.23, 4.40)	1,080	2.26 (–0.16, 4.67)
Model 4^*a*^	1,402	2.36 (0.23, 4.48)	1,042	2.30 (–0.16, 4.76)
Model 5^*a*^	1,302	3.02 (0.82, 5.22)	964	3.26 (0.70, 5.82)
Model 6^*a*^		NA	964	3.68 (0.98, 6.38)
Note: NA, not applicable. ^***a***^Model 2 was adjusted for sex, age (centered), and sex–age interaction; model 3 was additionally adjusted for educational level, smoking status at SAPALDIA2 (S2), smoking pack-years between S2 and SAPALDIA3 (S3) (centered), (smoking pack-years between S2 and S3)^2^, BMI at S2 (centered), (BMI at S2)^2^, BMI at S3 (centered) and (BMI at S3)^2^; model 4 was additionally adjusted for exposure to environmental tobacco smoke, alcohol intake, and physical activity (all in S3); model 5 was additionally adjusted for diabetes in S2, systolic blood pressure in S2 and S3 (centered), HDL cholesterol in S2 (centered), creatinine in S2 (centered), and intake of antihypertensive and lipid modifier medication in S3; model 6 (applied to nonmovers only) was additionally adjusted for exposure to night-time traffic noise at the residential address reported in S2. ^***b***^Average of the 365 days before the CIMT examination date. ^***c***^Exposure estimated for the 2011–2012 period. Sources of PM_2.5_ are expressed as a score derived from principal-component factor analysis (Aguilera et al. 2015).				

The comparison between effect estimates in the main model (model 3) with and without additional adjustment for PNC is reported in Table 3. For all pollutants except LDSA, effect estimates decreased after adjustment for PNC, the decrease being larger for PM_10_. The effect estimate for PNC was stronger in the model with the crustal factor of PM_2.5_, and negative in the model including LDSA. However, none of the associations in the two-pollutant model were statistically significant. Given the high correlations among pollutants and the stronger effect observed for LDSA as compared to PNC, models with the residuals obtained from regressing PNC and LDSA estimates against the other pollutants were also explored as an attempt to assess the independent contribution of PNC and LDSA separately to the association with CIMT. Effects were larger for LDSA than for PNC, but none of the associations were statistically significant (see Table S3).

**Table 3:** Estimated percent change in CIMT (95% CI) associated with an interdecile range (10th to 90th percentile) increase in air pollution exposures, in the main model and in a two-pollutant model adjusted for PNC exposure.

Exposure^*a*^	Main model^*b*^		Two-pollutant model^*b*^		PNC estimate in two-pollutant model^*c*^	
	*n*	% change (95% CI)	*n*	% change (95% CI)	*n*	% change (95% CI)
PM_10_ last year (5.5 μg/m^3^)	1,500	1.58 (–0.30, 3.47)	1,447	–0.05 (–4.30, 4.20)	1,447	2.13 (–2.31, 6.57)
PM_2.5_ last year (4.2 μg/m^3^)	1,500	2.10 (0.04, 4.16)	1,447	1.73 (–2.67, 6.13)	1,447	0.63 (–3.60, 4.86)
Vehicular source of PM_2.5_^*d*^	1,503	1.67 (–0.13, 3.48)	1,449	1.27 (–2.21, 4.74)	1,449	0.87 (–2.97, 4.72)
Crustal source of PM_2.5_^*d*^	1,503	–0.58 (–3.95, 2.79)	1,449	–1.53 (–4.99, 1.93)	1,449	3.35 (–0.20, 6.90)
LDSA (30.5 μm^2^/cm^3^)	1,449	2.32 (0.23, 4.40)	1,449	3.41 (–3.65, 10.46)	1,449	–1.11 (–8.00, 5.78)
PNC (12,639 particles/cm^3^)	1,449	2.06 (0.03, 4.10)		NA		NA
Note: NA, not applicable. ^***a***^Two-pollutant model for PM_10_ and PM_2.5_ exposure between SAPALDIA2 (S2) and SAPALDIA3 (S3) was not fitted due to the shorter time window of PNC exposure. ^***b***^Main model is adjusted for sex, age (centered), sex–age interaction, educational level, smoking status at S2, smoking pack-years between S2 and S3 (centered), smoking pack-years between S2 and S3^2^, BMI at S2 (centered), (BMI at S2)^2^, BMI at S3 (centered) and (BMI at S3)^2^. The two-pollutant model is additionally adjusted for PNC (interdecile range increase). ^***c***^PNC estimate based on a two-pollutant model with adjustment for the other PM exposure term shown in the first column. ^***d***^Sources of PM_2.5_ are expressed as a score derived from principal-component factor analysis (Aguilera et al. 2015).						

**Figure 1:**
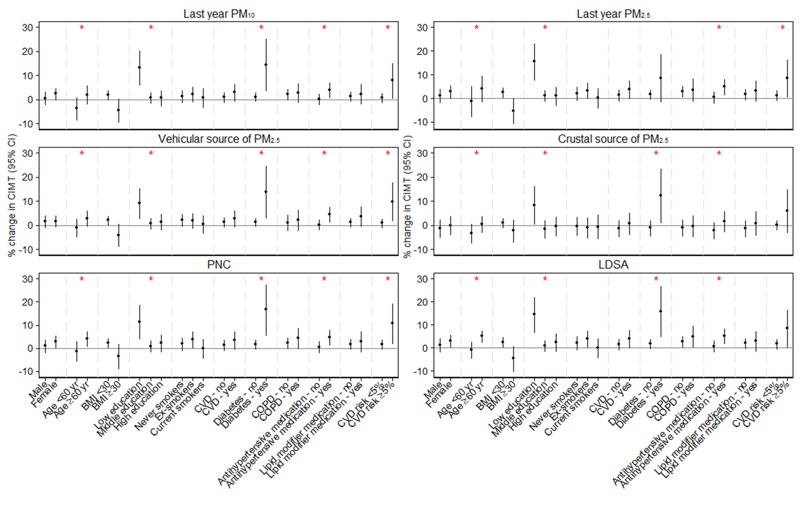
Estimated percent change in CIMT (95% CI) associated with an interdecile range increase in exposure estimates within subgroups of *a priori* selected covariates. Associations are adjusted for sex, age (centered), sex–age interaction, educational level, smoking status at SAPALDIA2 (S2), smoking pack-years between S2 and SAPALDIA3 (S3) (centered), (smoking pack-years between S2 and S3)^2^, BMI at S2 (centered), (BMI at S2)^2^, BMI at S3 (centered) and (BMI at S3)^2^. Red asterisk indicates a statistically significant effect modification by the covariate.

Associations stratified for *a priori* selected potential effect modifiers are illustrated in Figure 1. Effects were generally stronger in subjects who were older, nonobese, had lower educational level, were diabetics, used antihypertensive medication, or had higher CVD risk in SAPALDIA2. The differences in magnitude observed between strata were particularly large for educational level and diabetes.

Associations between exposure estimates and CIMT > 75th percentile showed similar result patterns (see Table S4), but were only statistically significant in a consistent manner across models for the vehicular source of PM_2.5_ among nonmovers.

The sensitivity analysis comparing the effect estimates using CIMT measurements from dynamic and static reading methods revealed almost no differences in effect estimates between NATIVE clips and stills from DSBG (see Figure S2). The differences in effect estimates using image stills from the two reading centres were larger, and effects were generally higher when using image stills from Imagelabonline.

## Discussion

We found a positive and statistically significant association between residential exposure to three size fractions of PM and CIMT across four geographically diverse areas of the Swiss SAPALDIA cohort. An association with CIMT for the vehicular source but not for the crustal source of PM_2.5_ was found. Associations were generally stronger when restricted to participants who had not changed their residential address in the decade before CIMT assessment. The more clinically oriented outcome of presenting CIMT in the upper tail of the distribution showed comparable results, although associations were statistically significant only for the vehicular source of PM_2.5_ within the subset of nonmovers.

This is the first epidemiological study to investigate the effect of UFP on subclinical atherosclerosis. The interest relies on the greater atherogenic potential of UFP observed in animal studies (Araujo et al. 2008) and the current lack of air quality standards for UFP. We found, however, a similar change in CIMT for both PNC and LDSA as compared to PM_2.5_ mass. The two-pollutant model showed that associations between CIMT and PM_10_ were the most sensitive to adjustment for PNC and that LDSA seemed to have a stronger effect than PNC. Although these models should be interpreted with caution because of the high correlations and the differences in measurement error between pollutants, our results for LDSA are in line with the findings from toxicological studies (Brook et al. 2010) and support the use of this novel metric in epidemiological studies.

The adjusted change in CIMT associated with a 10 μg/m^3^ increase in PM_2.5_ exposure in the present study was similar to the 4.2% [95% confidence interval (CI): –0.2%, 8.9%]change reported for the same exposure contrast in a study conducted in Los Angeles among participants in two clinical trials without known CVD, diabetes or hypertension (Künzli et al. 2005). A German population-based cohort of participants 45–75 years old also used PM_10_ and PM_2.5_ estimates from dispersion models derived for the last year before the CIMT assessment, with very similar mean exposure levels as in SAPALDIA (Bauer et al. 2010). However, the association observed for PM_2.5_ [4.1% increase (95% CI: 1.7%, 6.5%) per 4.2 μg/m^3^ PM_2.5_] was almost twice as high as in the present study for the same exposure contrast. In contrast, associations for PM_10_ were lower and nonsignificant [1.8% increase (95% CI: –0.6%, 4.3%) per 6.7 μg/m^3^ PM_10_]. The combined estimate in the European ESCAPE meta-analysis of three population-based cohorts 25–75 years old and one cohort of participants > 60 years old with increased risk for CVD showed a 1.44% (95% CI: –1.3%, 4.2%) increase per 10 μg/m^3^ PM_2.5_, and a nonsignificant inverse association for PM_10_ (Perez et al. 2015). Using the same exposure assessment methodology as in ESCAPE, an association was found for PM_10_ [2.7% increase (95% CI: 0.2%, 5.5%) per 10 μg/m^3^ PM_10_] but not for PM_2.5_ in a sample of control subjects 35–65 years old from an acute coronary heart disease study in Taiwan (Su et al. 2015). The cross-sectional association for PM_2.5_ was also lower in the most recent analysis of the population-based MESA study, with an estimated 0.4% (95% CI: –3.4%, 4.2%) change in CIMT for a 10 μg/m^3^ increase in PM_2.5_ within cities (Adar et al. 2013). Overall, the observed differences in effect estimates could be partly explained by the different exposure assessment methods and study population characteristics.

Results showed a clearly stronger association for vehicular-specific PM_2.5_ as compared to the crustal source. However, given that LUR model performance was lower for the crustal source than for the vehicular one, these results could also be related to an increased measurement error of the crustal source. To our knowledge, the effect of specific PM_2.5_ components and sources on CIMT has only been investigated in the MESA study (Kim et al. 2014). The analysis was focused on elemental carbon and organic carbon as markers of combustion sources, silicon as marker of crustal dust, and sulfur as indicator of sulfate (secondary aerosol). Strongest effects were found for organic carbon and sulfur, supporting the hypothesis of a different relative toxicity of various particle constituents.

We found slightly higher effect estimates for PM_10_ and PM_2.5_ exposures during the year prior to the CIMT assessment as compared to the time-weighted average exposures between SAPALDIA2 and SAPALDIA3 (see Table S5 and Figure S3). As expected, restricting the sample to nonmovers had little impact on the effect estimates of the time-weighted averages, as they were derived taking residential changes into account. However, effect estimates for exposures during the year prior to the CIMT assessment increased within this subsample, which suggests that the effect in the whole sample was attenuated due to potential exposure misclassification among movers. The finding of a weaker effect of longer-term PM exposure could be explained by several factors, including a potentially higher measurement error or a possibly reversible, rather than cumulative, effect of long-term exposure.

Most previous studies have reported differences in the association between air pollution and CIMT among population subgroups, however patterns have not been consistent (Adar et al. 2013; Bauer et al. 2010; Gan et al. 2014; Künzli et al. 2005; Perez et al. 2015; Rivera et al. 2013; Su et al. 2015). In the present study, the largest subgroup differences were found for educational level and diabetes. Although the subgroup of diabetics was small, results are consistent with previous research suggesting a greater susceptibility for cardiovascular effects of air pollution among persons with conditions linked to chronic inflammation such as diabetes, obesity and hypertension (Dubowsky et al. 2006). The larger change in CIMT found among those with low education remained after further adjustment for CVD, diabetes and COPD. But it is still conceivable that low socio-economic status captures residual confounding arising from undiagnosed conditions, particularly diabetes or hypertension, as well as less healthy lifestyles. Replacing the individual educational level variable with a neighbourhood-level socioeconomic index removed the observed effect modification completely (results not shown). These results suggest that the effect modification by educational level is unlikely to be explained by other co-exposures at the neighbourhood level. The larger effect of air pollution estimates on CIMT observed in subjects at higher CVD risk in SAPALDIA2 also supports the hypothesis that clusters of cardiovascular risk factors interact with air pollution exposure in the long-term.

Research is need to investigate the atherogenic role of traffic-related night-time noise and its potential confounding effect in the association between air pollution and atherosclerosis (Künzli 2013). Two previous studies on air pollution and CIMT that evaluated the potential confounding effect of noise did not find an effect (Adar et al. 2013; Gan et al. 2014). Another study, however, found an independent association of both PM_2.5_ and night-time traffic noise with thoracic aortic calcification (Kälsch et al. 2014). In our study, adjustment for night-time traffic noise slightly increased the observed associations for all air pollution exposure estimates. This indicates some possible degree of confounding between noise and air pollution in our population. As discussed by Foraster et al. (2011), the correlations between these two traffic-related stressors are likely to be heterogeneous within and possibly across cities and regions.

Among the strengths of the SAPALDIA cohort are the detailed characterization of study participants in terms of air pollution exposure and relevant covariates, the representativeness of the Swiss general population and its prospective design. The influence of selective attrition on the estimated associations was also assessed using inverse probability weighting (IPW), which showed very minor impacts on effect estimates (data not shown). This is the first study on air pollution and CIMT using NATIVE clips analysed with a fully automated system, validated within SAPALDIA3. This method is less dependent on field worker and resulted in narrower confidence intervals, thus might be better for investigating small effect sizes as expected for air pollution. An important limitation of this study, however, is the high correlation among all exposure estimates, which is the consequence of the high spatial correlation between the pollutants measured during the SAPALDIA sampling campaigns, slightly enhanced by the smoothing inherent to the air pollution modelling. This makes it difficult to disentangle specific effects of single pollutants. Nevertheless, this study constitutes an innovative effort to investigate the atherogenic effect of long-term exposure to PM using different metrics (including the novel metric LDSA for the ultrafine size range) and different sources of PM_2.5_. Given the public health relevance of atherosclerosis and the ubiquity of air pollution exposure, further epidemiological studies are needed to disentangle the atherogenic effects of different particle sizes and constituents.

## Supplemental Material

(703 KB) PDF
