# Supplementary material for: Particulate Matter and Subclinical Atherosclerosis: Associations between Different Particle Sizes and Sources with Carotid Intima-Media Thickness in the SAPALDIA Study
Source: Environ Health Perspect. 2016 Jun 3;124(11):1700–6. doi: 10.1289/EHP161 (PMC5089877; doi:10.1289/EHP161)
Supplement: (703 KB) PDF [file EHP161.s001.acco.pdf]

Note to readers with disabilities: *EHP* strives to ensure that all journal content is accessible to all readers. However, some figures and Supplemental Material published in *EHP* articles may not conform to 508 standards due to the complexity of the information being presented. If you need assistance accessing journal content, please contact ehp508@niehs.nih.gov. Our staff will work with you to assess and meet your accessibility needs within 3 working days.

Supplemental Material

Particulate Matter and Subclinical Atherosclerosis: Associations between Different Particle Sizes and Sources with Carotid Intima-Media Thickness in the SAPALDIA Study

Inmaculada Aguilera, Julia Dratva, Seraina Caviezel, Luc Burdet, Eric de Groot, Regina E. Ducret-Stich, Marloes Eeftens, Dirk Keidel, Reto Meier, Laura Perez, Thomas Rothe, Emmanuel Schaffner, Arno Schmit-Trucksäss, Ming-Yi Tsai, Christian Schindler, Nino Künzli, and Nicole Probst-Hensch

Table of Contents

List of SAPALDIA investigators

Additional methodological details

Table S1. Characteristics of the SAPALDIA study subjects aged 50 years or older included in the study and those excluded

Figure S1. Scatter plots of log-transformed mean CIMT values by air pollutant

Table S2. Pearson correlation coefficients between air pollution exposure estimates

Table S3. Estimated percent change in CIMT (95% CI) associated with the residuals obtained from: (a) regressing PNC estimates against PM_{10}, $PM_{2.5}$ and LDSA estimates, and (b) regressing LDSA estimates against PM_{10}, $PM_{2.5}$ and PNC estimates

Table S4. Associations between an interdecile range increase in air pollution exposures and CIMT > cohort-specific 75th percentile

Figure S2. Comparative analysis of percent change in CIMT (95% CI) associated

with an interdecile range increase in exposure estimates using three sources of CIMT data from two reading centres

Table S5. Estimated percent change in CIMT (95% CI) associated with a 10 μg/m^3 increase in PM_{10} and $PM_{2.5}$ exposures

Figure S3. Estimated percent change in CIMT (95% CI) associated with a 10 μg/m^3 increase in PM_{10} and $PM_{2.5}$ exposures within subgroups of selected covariates

References

The SAPALDIA team

Study directorate: NM Probst-Hensch (PI; e/g); T Rochat (p), C Schindler (s), N Künzli (e/exp), JM Gaspoz (c)

Scientific team: JC Barthélémy (c), W Berger (g), R Bettschart (p), A Bircher (a), C Brombach (n), PO Bridevaux (p), L Burdet (p), Felber Dietrich D (e), M Frey (p), U Frey (pd), MW Gerbase (p), D Gold (e), E de Groot (c), W Karrer (p), F Kronenberg (g), B Martin (pa), A Mehta (e), D Miedinger (o), M Pons (p), F Roche (c), T Rothe (p), P Schmid-Grendelmeyer (a), D Stolz (p), A Schmidt-Trucksäss (pa), J Schwartz (e), A Turk (p), A von Eckardstein (cc), E Zemp Stutz (e).

Scientific team at coordinating centers: M Adam (e), I Aguilera (e/exp), S Brunner (s), D Carballo (c), S Caviezel (pa), I Curjuric (e), A Di Pascale (s), J Dratva (e), R Ducret (s), E Dupuis Lozeron (s), M Eeftens (exp), I Eze (e), E Fischer (g), M Foraster (e), M Germond (s), L Grize (s), S Hansen (e), A Hensel (s), M Imboden (g), A Ineichen (exp), A Jeong (g), D Keidel (s), A Kumar (g), N Maire (s), A Mehta (e), R Meier (exp), E Schaffner (s), T Schikowski (e), M Tsai (exp)

(a) allergology, (c) cardiology, (cc) clinical chemistry, (e) epidemiology, (exp) exposure, (g) genetic and molecular biology, (m) meteorology, (n) nutrition, (o) occupational health, (p) pneumology, (pa) physical activity, (pd) pediatrics, (s) statistics

Acknowledgments
The study could not have been done without the help of the study participants, technical and administrative support and the medical teams and field workers at the local study sites.
Local fieldworkers : Aarau: S Brun, G Giger, M Sperisen, M Stahel, Basel: C Bürli, C Dahler, N Oertli, I Harreh, F Karrer, G Novicic, N Wyttenbacher, Davos: A Saner, P Senn, R Winzeler, Geneva: F Bonfils, B Blicharz, C Landolt, J Rochat, Lugano: S Boccia, E Gehrig, MT Mandia, G Solari, B Viscardi, Montana: AP Bieri, C Darioly, M Maire, Payerne: F Ding, P Danieli A Vonnez, Wald: D Bodmer, E Hochstrasser, R Kunz, C Meier, J Rakic, U Schafroth, A Walder.

Administrative staff: N Bauer Ott, C Gabriel, R Gutknecht

Additional methodological details

<u>CIMT measurements</u>

Image reading was performed in two vascular labs by experienced readers following a defined reading protocol:

Ultrasound analyses at Imagelabonline: DICOM B-mode images were analysed using SAPALDIA Fukuda dedicated eTrack ultrasound image analysis software, a semi-automated quantitative and qualitative analysis software (de Groot et al 1998).

Ultrasound analyses at DSBG: Detailed information about ultrasound examination and evaluation process are described elsewhere (Caviezel et al 2013, Teynor et al 2012). Briefly, the CCA far wall of single B-mode images (stills) and sequential B-mode images (clips) were analysed with the validated DYnamic ARtery Analysis (DYARA) software and mean CIMT was measured in a standardized 1cm segment proximal to the bulb in one image (stills) or across at least one heart cycle (clips) (Teynor et al 2012). Reproducibility of mean CIMT was evaluated in a random sample of 165 SAPALDIA3 participants whereby stills and clips showed a coefficient of variation of 3.95% (3.45–4.44) and 3.98% (3.52–4.44) and an intraclass correlation coefficient of 0.89 (0.86–0.92) and 0.89 (0.87–0.93), respectively (Caviezel et al 2013).

Following the Mannheim Consensus, measurement regions containing plaques and images with insufficient quality were excluded from analysis (Touboul et al 2012).

<u>Potential confounders and effect modifiers</u>

The following covariate information was obtained from the computer-assisted personal interviews performed at SAPALDIA2 and 3 examinations: age, sex, educational level (low/middle/high), current and past smoking status (never smoker/former smoker/current smoker), pack-years of cigarettes smoked, exposure to environmental tobacco smoke during the year prior to the examination (yes/no), alcohol intake (several times per week/less than several times per week), moderate physical activity at SAPALDIA3 (sufficiently active/not sufficiently active) and change between SAPALDIA2 and 3 (inactive mantainer/relapser/adopter/active mantainer), doctor-diagnosed CVD (yes/no), and intake of antihypertensive and lipid-lowering medication (yes/no). We also obtained information on relevant co-morbidities in SAPALDIA2, namely diabetes (defined as having at least one of the following: self-reported doctor-diagnosed diabetes, intake of any anti-diabetic medication, non-fasting glucose levels > 11.1 mmol/L, or glycosylated hemoglobin C [HbA1c] > 6.5% or 48 mmol/mol) (Eze et al 2014), and chronic obstructive pulmonary disease (COPD, defined using the Global Initiative for Chronic Obstructive Lung Disease [GOLD] fixed cut-off criterion: ratio of forced expiratory volume in 1 s over forced vital capacity [FEV_1/FVC] < 0.70).

The following covariate information was obtained through health examinations in SAPALDIA2 and 3. Body mass index (BMI, kg/m^2) was calculated using weight and height measured in each examination. BP was measured twice in the sitting position after a 10-minute rest with an interval of at least 3 minutes, and the mean of both systolic and diastolic BP measurements was calculated. Non-fasting cardio-metabolic and renal function biomarkers (glucose, total cholesterol, LDL and HDL cholesterol, triglycerides and creatinine) were available for subjects in SAPALDIA2 only (Ackermann-Liebrich et al 2005).

We also considered traffic noise as a potential confounder. Annual averages of traffic noise (equivalent continuous A-weighted levels) during night (22:00 to 06:00) at the participants' residential addresses were obtained from the SonBase Swiss model (Swiss Federal Office for the Environment 2009). Noise exposure estimates were available for the residential addresses at the SAPALDIA2 examination only.

Statistical analysis

A set of predetermined variables was tested for effect modification in the main models, namely sex, age (≥ and < 60 years), BMI (≥ and < 30 kg/m^2), educational level, smoking status, moderate physical activity, CVD and other co-morbidities (diabetes, COPD), and medication (antihypertensive and lipid-lowering). We also evaluated whether the association between air pollution and CIMT depended on the CVD risk at SAPALDIA2. For this purpose, we calculated the European Society of Cardiology heart risk score (SCORE) algorithm estimating the 10-year risk of a fatal event (Conroy et al 2003), based on data from SAPALDIA2 (age, smoking status, BP and cholesterol, separately by sex) (Dratva et al 2014), and tested for effect modification across two levels of risk (≥ and < 5%). Statistical significance of effect modification was set at P-value < 0.10.

In clinical settings, the upper tail of the CIMT distribution has particular relevance. To investigate the association between air pollution exposure and the risk of having substantially thickened artery walls, the outcome was defined as CIMT above the 75th percentile of the cohort-specific predicted value according to sex, age and BMI. These analyses relied on mixed logistic models with random effects. Predicted CIMT values were derived using quantile regression in the subsample of subjects without CVD and diabetes in SAPALDIA2 (n=1100).

Because residential exposures to PNC, LDSA and source-specific $PM_{2.5}$ were estimated using the address at SAPALDIA3, a sensitivity analysis was performed excluding subjects who had changed their residential addresses between SAPALDIA2 and 3. We assume misclassification of the long-term exposure to be less of an issue among non-movers. It was within this subset of non-movers where we were able to also adjust for exposure to night-time traffic noise (estimated at the SAPALDIA2 address), assuming that the spatial differences in noise levels remained rather stable over the follow-up period.

We focused the analysis on the mean CIMT measurements obtained using the NATIVE clips from DSGB. For comparison purposes with previous studies on air pollution and CIMT, we also conducted a sensitivity analysis using CIMT measurements from the static reading method (NATIVE image stills from DSBG and DICOM B-Mode image stills from Imagelabonline).

Results from the mixed linear and logistic models are reported as percent change in CIMT and odds ratio (OR) of CIMT > 75th percentile, respectively, associated with an interdecile range (10th to 90th percentile) increase in air pollution exposure. For PM_{10} and $PM_{2.5}$, associations are also reported for a 10 µg/m^3 increase to allow for comparison of associations with exposures between SAPALDIA2 and 3 and exposures during the last year before the CIMT assessment (Table S5 and Figure S3).

Table S1. Characteristics of the SAPALDIA study subjects aged 50 years or older included in the study[a] and those excluded, presented as percent or mean ± standard deviation

**Characteristics**	**Subjects included (n=1503)**	**Subjects excluded (n=999)**	**p-value[b]**
Study area			<0.001
Basel	22.0	26.9	
Geneva	14.3	19.6	
Lugano	25.7	24.6	
Wald	38.0	28.8	
Age (yrs)	63.9±8.2	65.3±8.9	<0.001
Women	53.0	57.3	0.04
Educational status			<0.001
Low (primary education)	5.5	10.4	
Middle (secondary or vocational education)	64.2	65.2	
High (technical college or university)	30.3	24.3	
Smoking status in S2			0.12
Never smoker	43.6	39.7	
Former smoker	34.1	35.3	
Current smoker	22.2	25	
Smoking pack-years from S2 to S3	1.12±2.95	1.4±3.4	0.07
Exposed to ETS in the last year	12.8	15.3	0.08
Alcohol intake in S3, several times per week	44.7	44.2	0.84
Moderate physical activity category change between S2 and S3			0.11
Inactive mantainer	26.5	26.2	
Relapser	17.1	22.3	
Adopter	21.7	19	
Active mantainer	34.6	32.6	
Moderate physical activity in S3, sufficiently active	56.0	51.3	0.06
BMI in S2 (kg/m^2)	25.6±4.0	27.0±4.9	<0.001
BMI in S3 (kg/m^2)	26.3±4.4	28.2±5.6	<0.001
Systolic blood pressure in S2 (mm Hg)	127.8±18.8	130.8±19.9	0.002
Systolic blood pressure in S3 (mm Hg)	134.9±18.9	135.8±19.2	0.24
Total cholesterol in S2 (mg/dL)	236.5±41.4	238.8±42.6	0.36
HDL in S2 (mg/dL)	59.1±17.4	58.3±18.2	0.30
Triglycerides in S2 (mg/dL)	155.1±97.6	176.8±124.4	<0.001
Creatinine in S2 (mg/dL)	0.99±0.14	0.99±0.14	0.90
CVD risk SCORE[c] in S2, 10-yr risk ≥ 5%	5.7	9.0	<0.001

Doctor-diagnosed CVD in S2	24.6	31.5	<0.001
Diabetes[d] in S2	3.9	6.6	<0.001
COPD[e] in S2	22.1	25.0	0.17
Antihypertensive medication in S3	33.1	35.0	0.39
Lipid modifier medication in S3	20.1	20.8	0.72

[a] Subjects with valid air pollution exposure estimates, who participated in both SAPALDIA2 (S2) and SAPALDIA3 (S3), had CIMT measurements, and complete information in covariates included in the main model.

[b] P-value of the difference between the two groups using Chi-square or Mann-Withney test.

[c] Score for a 10-year risk of a fatal event [13].

[d] Defined as having at least one of the following: self-reported doctor-diagnosed diabetes, intake of any anti-diabetic medication, glucose levels > 11.1 mmol/L, or HbA1c > 6.5% or 48 mmol/mol.

[e] Defined as $FEV_1/FVC < 0.70$.

Figure S1. Scatter plots of log-transformed mean CIMT values by air pollutant

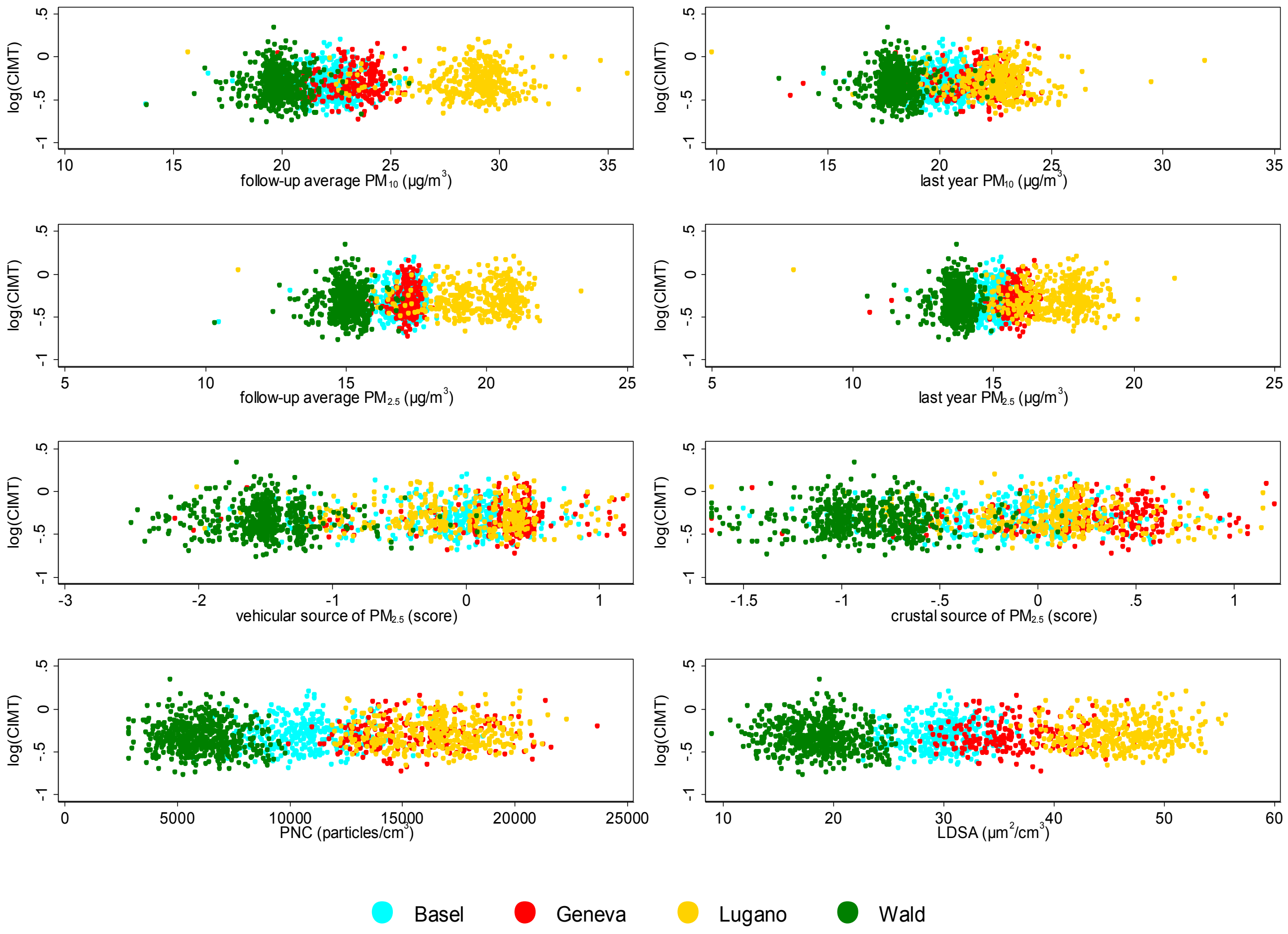

Table S2. Pearson correlation coefficients between air pollution exposure estimates

	PM_{10} between S2 and S3	PM_{10} last year	$PM_{2.5}$ between S2 and S3	$PM_{2.5}$ last year	$PM_{2.5}$ - Vehicular source	$PM_{2.5}$ - Crustal source	PNC	LDSA
PM_{10} between S2 and S3	1							
PM_{10} last year[a]	0.90	1						
$PM_{2.5}$ between S2 and S3	0.97	0.89	1					
$PM_{2.5}$ last year[a]	0.93	0.93	0.96	1				
$PM_{2.5}$ - Vehicular source	0.72	0.85	0.80	0.82	1			
$PM_{2.5}$ - Crustal source	0.73	0.91	0.75	0.78	0.89	1		
PNC	0.85	0.90	0.86	0.88	0.86	0.83	1	
LDSA	0.93	0.91	0.94	0.94	0.82	0.79	0.96	1

[a] Average of the 365 days before the CIMT examination date.

Table S3. Estimated percent change in CIMT (95% CI) associated with the residuals obtained from: (a) regressing PNC estimates against PM_{10}, $PM_{2.5}$ and LDSA estimates, and (b) regressing LDSA estimates against PM_{10}, $PM_{2.5}$ and PNC estimates.

**Model**[a]	**N**	**% change (95% CI)**
Residuals of PNC vs. PM_{10} last year[b]	1447	1.55 (-3.04, 6.15)
Residuals of PNC vs. $PM_{2.5}$ last year[b]	1447	0.27 (-4.23, 4.78)
Residuals of PNC vs. LDSA	1449	-0.13 (-8.01, 7.74)
Residuals of LDSA vs. PM_{10} last year[b]	1447	2.42 (-3.09, 7.93)
Residuals of LDSA vs. $PM_{2.5}$ last year[b]	1447	0.43 (-5.79, 6.66)
Residuals of LDSA vs. PNC	1449	1.42 (-7.35, 10.20)

[*a]Adjusted for sex, age (centered), sex-age interaction, educational level, smoking status at S2, smoking pack-years between S2 and S3 (centered), (smoking pack-years between S2 and S3)^2, BMI at S2 (centered), (BMI at S2)^2, BMI at S3 (centered), and (BMI at S3)^2.

[b] Average of the 365 days before the CIMT examination date.

Table S4. Associations (expressed as ORs with 95% CIs) between an interdecile range increase in air pollution exposures and CIMT > cohort-specific 75th percentile[a], for the entire sample and for non-movers

	All subjects		Non-movers	
Exposure (P90-P10 increase)	N	OR (95% CI)	N	OR (95% CI)
**Average PM_{10} between S2 and S3 (increase of 10 $\mu g/m^3$)**				
Model 1 (crude)	1491	1.23 (0.88, 1.72)	1101	1.40 (0.77, 2.54)
Model 2 (main)[b]	1491	1.17 (0.86, 1.60)	1101	1.35 (0.72, 2.51)
Model 3[b]	1443	1.19 (0.87, 1.64)	1063	1.41 (0.73, 2.74)
Model 4[b]	1340	1.22 (0.84, 1.79)	983	1.46 (0.75, 2.85)
Model 5[b]		n/a	983	1.64 (0.74, 3.64)
**Average PM_{10} of the last year[c] (increase of 5.5 $\mu g/m^3$)**				
Model 1 (crude)	1500	1.22 (0.92, 1.62)	1102	1.50 (0.92, 2.45)
Model 2 (main)[b]	1500	1.17 (0.87, 1.56)	1102	1.44 (0.87, 2.38)
Model 3[b]	1452	1.18 (0.88, 1.59)	1064	1.50 (0.90, 2.50)
Model 4[b]	1348	1.20 (0.87, 1.65)	984	1.51 (0.91, 2.52)
Model 5[b]		n/a	984	1.66 (0.94, 2.95)
**Average $PM_{2.5}$ between S2 and S3 (increase of 5.6 $\mu g/m^3$)**				
Model 1 (crude)	1491	1.30 (0.94, 1.79)	1101	1.48 (0.85, 2.57)
Model 2 (main)[b]	1491	1.23 (0.89, 1.70)	1101	1.43 (0.80, 2.53)
Model 3[b]	1443	1.25 (0.90, 1.74)	1063	1.50 (0.82, 2.76)
Model 4[b]	1340	1.31 (0.92, 1.87)	983	1.59 (0.85, 2.99)
Model 5[b]		n/a	983	1.74 (0.86, 3.52)
**Average $PM_{2.5}$ of the last year[c] (increase of 4.2 $\mu g/m^3$)**				
Model 1 (crude)	1500	1.23 (0.89, 1.69)	1102	1.48 (0.85, 2.59)
Model 2 (main)[b]	1500	1.15 (0.84, 1.58)	1102	1.39 (0.79, 2.44)
Model 3[b]	1452	1.17 (0.85, 1.62)	1064	1.45 (0.81, 2.61)
Model 4[b]	1348	1.20 (0.89, 1.82)	984	1.49 (0.82, 2.71)
Model 5[b]		n/a	984	1.58 (0.83, 3.03)
**Vehicular source of $PM_{2.5}$, biennial average[d]**				
Model 1 (crude)	1503	1.28 (0.97, 1.69)	1102	**1.45 (1.04, 2.00)**
Model 2 (main)[b]	1503	1.25 (0.94, 1.65)	1102	**1.40 (1.00, 1.97)**
Model 3[b]	1455	1.28 (0.96, 1.71)	1064	**1.49 (1.05, 2.11)**
Model 4[b]	1351	1.33 (0.98, 1.81)	984	**1.62 (1.12, 2.35)**
Model 5[b]		n/a	984	**1.79 (1.20, 2.66)**
**Crustal source of $PM_{2.5}$, biennial average[d]**				
Model 1 (crude)	1503	1.18 (0.88, 1.58)	1102	**1.48 (1.02, 2.15)**
Model 2 (main)[b]	1503	1.14 (0.84, 1.53)	1102	1.44 (0.97, 2.13)
Model 3[b]	1455	1.15 (0.85, 1.56)	1064	1.50 (0.99, 2.23)
Model 4[b]	1351	1.15 (0.83, 1.60)	984	1.54 (0.98, 2.43)
Model 5[b]		n/a	984	**1.68 (1.03, 2.73)**
**PNC, biennial average[d] (increase of 12,639 particles/cm^3)**				
Model 1 (crude)	1449	1.17 (0.86, 1.59)	1080	1.21 (0.76, 1.90)
Model 2 (main)[b]	1449	1.13 (0.83, 1.54)	1080	1.15 (0.72, 1.85)
Model 3[b]	1402	1.17 (0.85, 1.62)	1042	1.25 (0.76, 2.05)
Model 4[b]	1302	1.28 (0.90, 1.80)	964	1.38 (0.82, 2.34)

Model 5[b]		n/a	964	1.54 (0.84, 2.80)
**LDSA, biennial average[d] (increase of 30.5 $\mu m^2/cm^3$)**				
Model 1 (crude)	1449	1.20 (0.88, 1.64)	1080	1.25 (0.77, 2.03)
Model 2 (main)[b]	1449	1.14 (0.83, 1.56)	1080	1.17 (0.70, 1.94)
Model 3[b]	1402	1.18 (0.85, 1.63)	1042	1.25 (0.73, 2.16)
Model 4[b]	1302	1.24 (0.88, 1.77)	964	1.31 (0.73, 2.34)
Model 5[b]		n/a	964	1.42 (0.74, 2.71)

[a] Cohort-specific 75th percentile modeled by quantile regression as a function of sex, age and BMI in subjects without cardiovascular disease and diabetes.

[b] Model 2 was adjusted for educational level, smoking status at S2 and smoking pack-years between S2 and S3 (centered); model 3 was additionally adjusted for exposure to environmental tobacco smoke, alcohol intake and physical activity (all in S3); model 4 was additionally adjusted for diabetes in S2, systolic blood pressure in S2 and S3 (centered), HDL cholesterol in S2 (centered), creatinine in S2 (centered), and intake of antihypertensive and lipid modifier medication in S3; model 5 (applied to non-movers only) was additionally adjusted for exposure to night-time traffic noise at the residential address reported in S2.

[c] Average of the 365 days before the CIMT examination date.

[d] Exposure estimated for the 2011–2012 period. Sources of $PM_{2.5}$ are expressed as a score derived from principal-component factor analysis. [3]

Figure S2. Comparative analysis of percent change in CIMT (95% CI) associated with an interdecile range increase in exposure estimates using three sources of CIMT data from two reading centres. Model 1 is crude effects model. Model 3 (main) is adjusted for sex, age (centered), sex-age interaction, educational level, smoking status at S2, smoking pack-years between S2 and S3 (centered), (smoking pack-years between S2 and S3)^2, BMI at S2 (centered), (BMI at S2)^2, BMI at S3 (centered), and (BMI at S3)^2. Model 5 is additionally adjusted for exposure to environmental tobacco smoke in S3, alcohol intake in S3, physical activity in S3, diabetes in S2, systolic blood pressure in S2 and S3 (centered), HDL cholesterol in S2 (centered), creatinine in S2 (centered), and intake of antihypertensive and lipid modifier medication in S3. All associations for image stills from Imagelabonline are also adjusted by field worker.

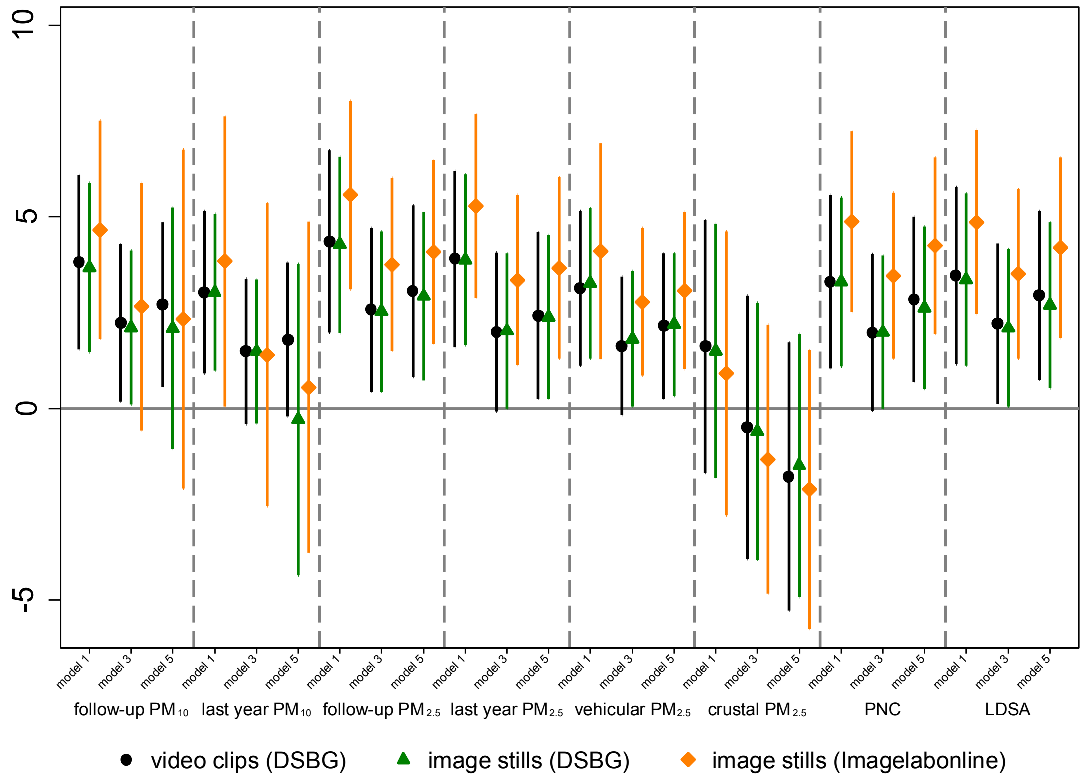

Table S5. Estimated percent change in CIMT (95% CI) associated with a 10 μg/m^3 increase in PM_{10} and $PM_{2.5}$ exposures, for the entire sample and for non-movers.

	All subjects		Non-movers	
	N	% change (95% CI)	N	% change (95% CI)
**Average PM_{10} between S2 and S3 (10 μg/m^3 increase)**				
Model 1 (crude)	1491	4.00 (1.70, 6.29)	1101	4.37 (1.64, 7.09)
Model 3 (main)[a]	1491	2.34 (0.28, 4.40)	1101	2.23 (-0.21, 4.68)
**Average PM_{10} of the last year[b] (10 μg/m^3 increase)**				
Model 1 (crude)	1500	5.52 (1.70, 9.33)	1102	7.24 (2.61, 11.87)
Model 3 (main)[a]	1500	2.86 (-0.55, 6.28)	1102	4.02 (-0.13, 8.18)
**Average $PM_{2.5}$ between S2 and S3 (10 μg/m^3 increase)**				
Model 1 (crude)	1491	7.98 (3.76, 12.20)	1101	8.45 (3.45, 13.45)
Model 3 (main)[a]	1491	4.68 (0.89, 8.47)	1101	4.58 (0.08, 9.08)
**Average $PM_{2.5}$ of the last year[b] (10 μg/m^3 increase)**				
Model 1 (crude)	1500	9.43 (3.99, 14.86)	1102	11.06 (4.51, 17.62)
Model 3 (main)[a]	1500	4.96 (0.09, 9.83)	1102	6.07 (0.18, 11.96)

[a] Adjusted for sex, age (centered) and sex-age interaction, educational level, smoking status at S2, smoking pack-years between S2 and S3 (centered), (smoking pack-years between S2 and S3)^2, BMI at S2 (centered), (BMI at S2)^2, BMI at S3 (centered) and (BMI at S3)^2.

[b] Average of the 365 days before the CIMT examination date.

Figure S3. Estimated percent change in CIMT (95% CI) associated with a 10 μg/m^3 increase in PM_{10} and $PM_{2.5}$ exposures within subgroups of selected covariates. Associations are adjusted for sex, age (centered), sex-age interaction, educational level, smoking status at S2, smoking pack-years between S2 and S3 (centered), (smoking pack-years between S2 and S3)^2, BMI at S2 (centered), (BMI at S2)^2, BMI at S3 (centered), and (BMI at S3)^2. Red asterisk indicates a statistically significant effect modification by the covariate.

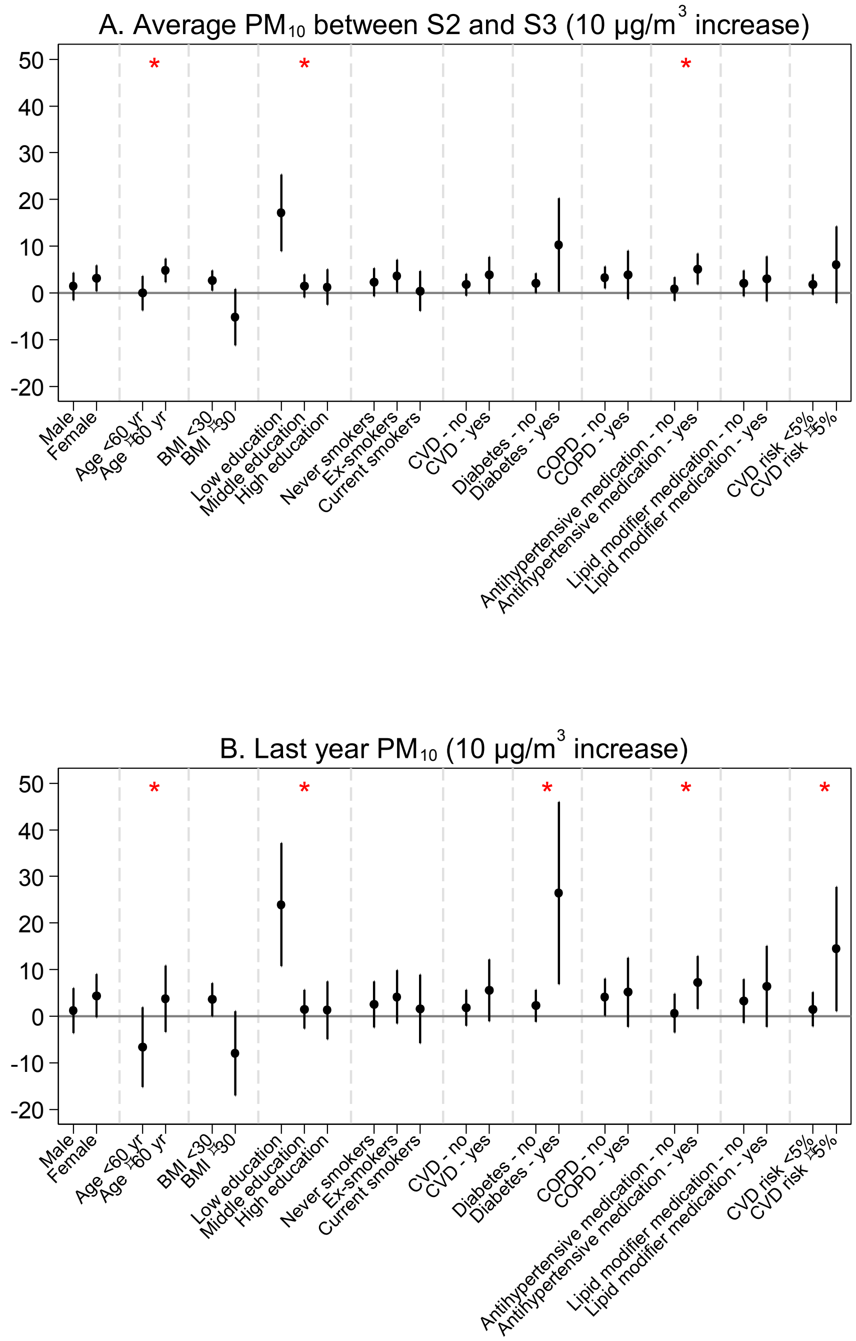

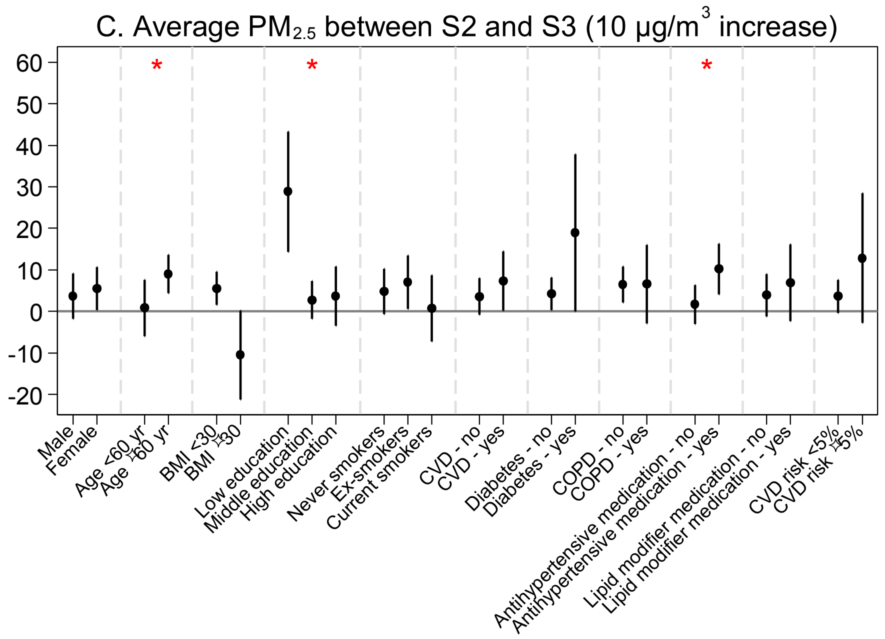
C. Average PM2.5 between S2 and S3 (10 μg/m3 increase)
60
50
40
30
20
10
0
-10
-20
*
*
*
Male
Female
Age <60 yr
Age ≥60 yr
BMI <30
BMI ≥30
Low education
Middle education
High education
Never smokers
Ex-smokers
Current smokers
CVD - no
CVD - yes
Diabetes - no
Diabetes - yes
COPD - no
COPD - yes
Antihypertensive medication - no
Antihypertensive medication - yes
Lipid modifier medication - no
Lipid modifier medication - yes
CVD risk <5%
CVD risk ≥5%

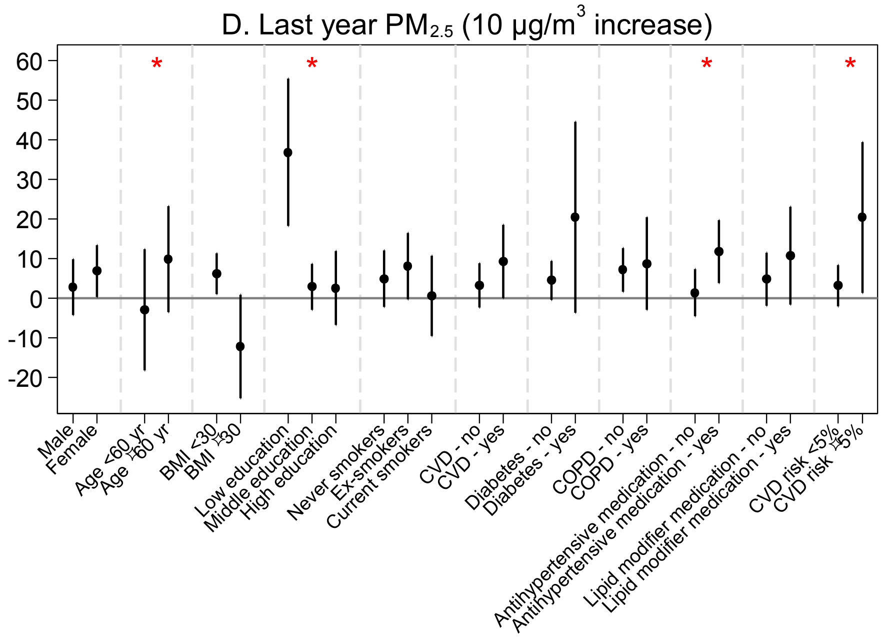
D. Last year PM2.5 (10 μg/m3 increase)
60
50
40
30
20
10
0
-10
-20
*
*
*
*
Male
Female
Age <60 yr
Age ≥60 yr
BMI <30
BMI ≥30
Low education
Middle education
High education
Never smokers
Ex-smokers
Current smokers
CVD - no
CVD - yes
Diabetes - no
Diabetes - yes
COPD - no
COPD - yes
Antihypertensive medication - no
Antihypertensive medication - yes
Lipid modifier medication - no
Lipid modifier medication - yes
CVD risk <5%
CVD risk ≥5%

References

Ackermann-Liebrich U, Kuna-Dibbert B, Probst-Hensch NM, et al. 2005. Follow-up of the Swiss Cohort Study on Air Pollution and Lung Diseases in Adults (SAPALDIA 2) 1991-2003: methods and characterization of participants. Soz Praventivmed 50:245–263.

Caviezel S, Dratva J, Schaffner E, et al. 2013. Variability and reproducibility of carotid structural and functional parameters assessed with transcutaneous ultrasound - results from the SAPALDIA Cohort Study. Atherosclerosis 231:448–455.

Conroy RM, Pyorala K, Fitzgerald AP, et al. 2003. Estimation of ten-year risk of fatal cardiovascular disease in Europe: the SCORE project. Eur Heart J 24:987–1003.

de Groot E, Zwinderman AH, van der Steen AF et al. 1998. Variance components analysis of carotid and femoral intima-media thickness measurements. REGRESS Study Group, Interuniversity Cardiology Institute of The Netherlands, Utrecht, The Netherlands. Regression Growth Evaluation Statin Study. Ultrasound Med Biol 24:825–832.

Dratva J, Caviezel S, Schaffner E, et al. 2014. Early detection of subjects at risk for vascular remodelling - results from the Swiss population-based study SAPALDIA. Swiss Med Wkly 144:w14052.

Eze IC, Schaffner E, Zemp E, et al. 2014. Environmental tobacco smoke exposure and diabetes in adult never-smokers. Environ Health 13: 74.

Teynor A, Caviezel S, Dratva J, Kunzli N, Schmidt-Trucksass A. 2012. An automated, interactive analysis system for ultrasound sequences of the common carotid artery. Ultrasound Med Biol 38: 1440–50.

Touboul PJ, Hennerici MG, Meairs S, et al. 2012. Mannheim carotid intima-media thickness and plaque consensus (2004-2006-2011). An update on behalf of the advisory board of the 3rd, 4th and 5th watching the risk symposia, at the 13th, 15th and 20th European Stroke Conferences, Mannheim, Germany, 2004, Brussels, Belgium, 2006, and Hamburg, Germany, 2011. Cerebrovasc Dis 34:290–296.

Swiss Federal Office for the Environment 2009: Noise Pollution in Switzerland. Results of the SonBase National Noise Monitoring Programme. State of the Environment No. 0907. Bern, Switzerland. 61 pp.
